# Integrons in the Intestinal Microbiota as Reservoirs for Transmission of Antibiotic Resistance Genes

**DOI:** 10.3390/pathogens3020238

**Published:** 2014-03-31

**Authors:** Anuradha Ravi, Ekaterina Avershina, Jane Ludvigsen, Trine M. L’Abée-Lund, Knut Rudi

**Affiliations:** 1 Norwegian University of Life Sciences, Chemistry, Biotechnology and Food science department (IKBM), Campus Ås, Ås 1432, Norway; E-Mails: anuradha.ravi@nmbu.no (A.R.); ekaterina.avershina@nmbu.no (E.A.); jane.ludvigsen@nmbu.no (J.L.); 2 Norwegian University of Life Sciences, Department of Food Safety and Infection Biology, Campus Adamstuen, Oslo 0454, Norway; E-Mail: trine.labee-lund@nmbu.no

**Keywords:** microbiota, antibiotic resistance genes, integrons, commensal flora

## Abstract

The human intestinal microbiota plays a major beneficial role in immune development and resistance to pathogens. The use of antibiotics, however, can cause the spread of antibiotic resistance genes within the resident intestinal microbiota. Important vectors for this are integrons. This review therefore focuses on the integrons in non-pathogenic bacteria as a potential source for the development and persistence of multidrug resistance. Integrons are a group of genetic elements which are assembly platforms that can capture specific gene cassettes and express them. Integrons in pathogenic bacteria have been extensively investigated, while integrons in the intestinal microbiota have not yet gained much attention. Knowledge of the integrons residing in the microbiota, however, can potentially aid in controlling the spread of antibiotic resistance genes to pathogens.

## 1. Global Spread of Antibiotic Resistance Genes

Antimicrobial agents have been used for the treatment of many bacterial infections in humans. They have been used to cure bacterial meningitis, endocarditis, skin infections, urinary tract infections and many more diseases [1,2]. Unfortunately, the widespread use of antibiotics has led to the spread of antibiotic resistance (AR) genes. This is a growing and serious problem that has raised several health concerns over the past few years [3,4,5].

The approval process for antibiotic use has become more stringent in the recent years due to the emergence of resistant strains [6]. However, in most developing countries, antibiotic treatment is not yet regulated and is often misused. Therefore, many of the so-called superbugs or multi-drug resistant strains have emerged from developing countries and spread to the rest of the world. For example, carbapenem-resistant *Klebsiella pneumoniae* strain which is resistant to most of the commonly used antibiotics has spread its resistance gene to the other members of the *Enterobacteriaceae* family such as *Escherichia coli* [7]. The New Delhi metallo β-lactamase (NDM) resistance gene arose in India, though it was first isolated in Sweden [6]. It has been shown that most of the current human-associated microbes, irrespective of the source of isolation, contain at least one acquired resistance gene to one or more antibiotics [8,9].

The increase of antibiotic resistance is an emerging problem not only for humans, but also for farm animals [10] and insects [11]. For example, in some countries where the antibiotic usage is not thoroughly regulated, the overuse of antibiotics in farm animals has become a looming threat to public health. Antibiotics are given to the animal in low doses throughout its life as growth promoters [10,12] that increase feed efficiency. Due to structural similarities between antibiotics used in animals and humans, the spread of genes from treated farm animals can also confer cross-resistance towards the human antibiotics [6]. Therefore, the general dissemination of antibiotic resistance must be considered on a global scale [3,13,14].

Until recently, bacterial pathogens have been the prime focus for the spread of AR genes. When Smillie and colleagues analyzed the rate of horizontal gene transfer (HGT) in bacteria from different environments [15], they concluded that regardless of the phylogenetic relatedness, human-associated bacteria show highly increased HGT rate (up to 25-fold) compared to non-human associated bacteria. Moreover, they also showed that AR genes were observed in around 50% of HGT events in the human gut. In another study, which addressed the gut-associated resistome of healthy infants [16], Moore and colleagues observed diverse AR-conferring sequences in pediatric fecal microbiota, many of which had low identity to sequences from known organisms, suggesting a broad range of hosts. Taking into account such surprisingly high HGT rates, AR genes diversity and scarcity of their taxonomical identity, it becomes reasonable to assume that gut microbiota may also play a crucial role in persistence and spread of antibiotic resistance.

## 2. Gene Transfer Mechanisms

HGT, including the transmission of AR genes are of prime importance in bacterial evolution, [8,17,18], since bacterial genomes have a remarkable ability to take up and express new genes [19]. The elements that contribute to the genome plasticity are mobile genetic elements (MGE) (transposons, integrons, and conjugative transposons). MGEs are segments of DNA that encode proteins which mediate the transfer of DNA within or between bacterial cells [20,21]. Recent studies on MGEs have shown them to play important roles in the spread of resistance genes, biotransformation of xenobiotics and bacterial symbiosis [21].

Most common HGT mechanisms in bacteria are (i) *conjugation*—transfer of plasmids and transposons through direct cell-to-cell contact, (ii) *transformation*—incorporation and expression of exogenous genetic material from its surroundings and (iii) *transduction*—bacteriophage induction of foreign genes [22]. Particularly, conjugation has been extensively studied. It is often regarded as the major reason for the spread of antibiotic resistance in closely associated bacteria [23,24,25]. It can induce a SOS system that can impact inter- and intra-species integration and recombination [23,26]. Transfer of drug resistance genes via conjugation has been shown to take place between distantly related bacteria in both lab standardized and in natural mimicking environments as well as *in vivo*. Kruse and Sørum [27] showed conjugal transfer of multi drug resistance plasmids between distantly related bacteria in natural micro-environments. The transfer of the plasmid from a bacterial pathogen of human, fish and animal origin to a susceptible strain from another ecological niche was able to occur within one hour despite suboptimal conditions and no selective pressure. Lester *et al.* have shown intra-species conjugal transfer of resistance genes in a transposon from multi-drug-resistant *Enterococcus faecium* to plasmid-free *Enterococcus faecium* through *in vivo* experiments in mice [28]. Another common mechanism of HGT among bacteria is transformation. Natural transformation was indicated in a recent study by Domingues *et al.* where genetic transfer of non-conjugative and incomplete mobile genetic elements (MGEs) was shown [29]. In another study, *Acinetobacter baylyi* was the model organism exposed to *Tn21* transposon from a *Salmonella* strain [30]. The model showed single inserts at different chromosomal locations in the bacteria which confirmed horizontal transfer by transformation. The final common mechanism for bacterial HGT is transduction, *i.e.*, gene transfer by bacteriophages. Since there is no apparent genetic signature, it is very difficult to identify these events [19,29,31]. Transduction in the GI tract maybe common, but unfortunately until now, there is lack of research in this area.

## 3. Integrons

**Figure 1:**
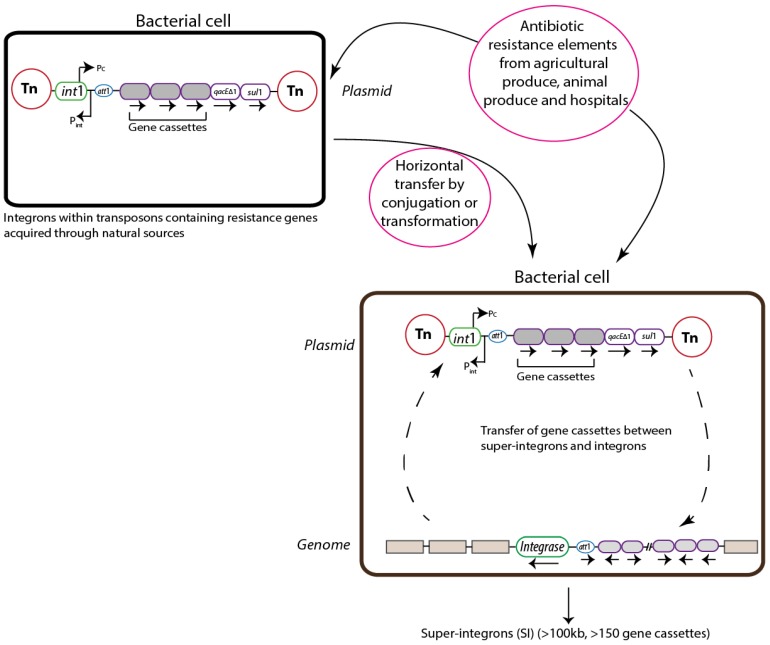
Transfer of antibiotic resistance (AR) genes through integrons: the figure gives a schematic representation of the transmission of integrons. Transposons (Tn) containing integrons can transfer into a bacterial cell from natural sources. The *int*1 and the *att*1 are responsible for the integration and attachment of the gene cassettes. The *qacE*Δ1 and *sul*1 genes encode resistance to quaternary ammonium compounds and sulfonamides, respectively. The grey areas represent the gene cassettes with varied functions. The grey boxes represent the genes that are outside to the super-integron. The P_int_ and P_c_ are promoters for the *integrase* (*int*1) gene and captured gene cassettes, respectively. Super-integrons may contain genes having their own promoters and may not rely on the integron for expression. The sizes of the figures do not relate to the gene sizes.

Integrons are platforms that help in the integration, assembly and expression of mobile promoterless genes referred to as gene cassettes [13,18,20,32]. Integrons are generally non-mobile and are often located on MGEs like transposons and plasmids that could serve as vehicles for the inter- and intra-species transmission of genes [29,33] (Figure 1). The gene cassettes in the integrons are mainly AR genes which are expressed by a common promoter that ensures the correct expression of these cassettes. A recent study demonstrated that most of the integrons that have been sequenced and characterized contain at least one acquired resistance gene [33]. The number of gene cassettes can vary in an integron but the highest number of gene cassettes observed so far is eight [20].

### 3.1. Classification

Integrons are classified based on sequence similarity. There are at least five classes of integrons with class I integrons being the most studied and characterized. They are reported on many gram-negative genera associated with the gut microbiota like *Acinetobacter* [34,35], *Escherichia coli* [36,37], *Salmonella* [38] and many more [33]. The class II integrons are often associated with *Tn7* family of transposons and also found in *Salmonella* [39] and *Shigella* [40]. Class III integrons are very similar to the other two classes but are related to the Tn*402* transposon [20]. The class IV and V have been identified in association with the development of trimethoprim resistance in *Vibrio* species [41,20].

### 3.2. Structure

In general, 3 key elements are required for integration. (i) The integrase gene which belongs to the tyrosine recombinase family that is involved in the integration of the gene cassettes at the attachment site [13,20,32,33]. (ii) The recombination site can be of two types depending on its location (1) on the integron: *att*1, primary site of attachment of the gene cassettes and (2) on the gene cassette itself: *att*C [33]. These sites are recognized by the integrase and are essential in recombination of the cassettes present in the integron. (iii) The outward-oriented promoter in the integrase which directs the transcription of the inserted gene cassettes, since they cannot express independently.

### 3.3. Evolution

Super-integrons are regarded as the ancient precursors of the present integrons [20]. Unlike integrons, the super-integrons are always chromosomally located. They are larger in size and carry more than 20 gene cassettes (cases of hundreds of gene cassettes have been described), in which many are of unknown function [20]. The gene cassettes in the super-integrons are not expressed by a common promoter, and can have their own promoters. These cassettes are not oriented in a stringent manner as in the class I integrons. The super-integrons were first identified on the chromosomes of *Vibrio cholera* [42], but have since then been identified in several other bacterial species [42,43,44].

### 3.4. Mobility

Various evidence exists that show integrons moving through bacterial species and transferring multidrug resistance properties [45,46]. Miller *et al.* [47] explained the role of the SOS responses from the bacterial cell that controls the expression of the integrase in site-specific recombination events. This response drives the positional regulation (site-specific recombination) of the gene cassettes in the integron: the closer the gene cassette is to the *att*1 site, the higher is its expression. The transfer of integrons through HGT is also likely at regions of close proximity between the bacterial species [47]. The emergence of multidrug resistant bacteria is mainly due to the close proximity of the drug and bacteria. Guo *et al.* demonstrated the diversity of class I integrons and gene cassettes in *Enterobacteriaceae,* as a pattern in hospital waste water [45]. Authors indicated that integrons generated new linkages for antibiotic resistance mostly at areas of high exposure to antimicrobial agents. Therefore, antimicrobial selective pressure plays a major role in acquiring resistance genes. Leverstein van-Hall *et al.* showed the persistence of integrons in a neurosurgery ward of a hospital where there was evidence of several patients carrying related multi-drug resistance integrons [46]. Most of the integrons detected were sourced to the general ward of the hospital. The patients in the study, contained same integrons within different bacterial strains and same integron genotype with different antibiotic resistance genes [46]. The authors hypothesized that the persistence of resistance genes was due to the structural association with other resistance genes.

The organization of integrons in a conjugative plasmid or transposons generally increase the genetic fitness of the bacteria [48]. The ancestral integrons may not have contained many resistance genes but with the increase in antibiotic usage, most integrons sequenced to date harbor the *qac* and *sul1* genes that confer resistance to biocides and sulfonamides respectively [49,50]. Therefore, with more integrons being characterized and sequenced, knowledge regarding the integron diversity is increasing.

## 4. Integrons in the Intestinal Microbiota as Reservoirs for AR Genes

The human microbiota is mainly composed of non-pathogenic bacteria. The gut microbiota is one of the most densely populated communities known so far with about 1000 distinct species co-existing in close proximity [51]. Historically, it has been assumed that the human gut is sterile during pre-natal development [52,53], though lately the results of several studies appear to challenge this notion [54,55,56]. During the first year of life, the human gut microbiota transitions through many compositional perturbations starting with aerobic bacteria which are outcompeted by anaerobes when oxygen levels are reduced as time passes [57]. Later on, development slows down [58] and then reaches the “adult-like-state” within the next two years [51]. In adulthood, bacterial cells in the gut outnumber human somatic cells by at least one log [59,60]. As a matter of fact, more than 50% of fecal mass accounts for bacteria [61]. It is not surprising that with such considerable densities, gut bacteria develop a strong cross-talk between various species [62,63]. This cross-talk may also lead to an increased exchange of genetic information throughout the commensal flora [15].

The intestinal microbiota is repeatedly challenged by antimicrobial pressures during the lifetime of the host. Although there has not yet been a major focus on non-pathogenic bacteria with respect to spread of AR resistance, integrons in the microbiota have been shown to be a reservoir for AR genes in several studies (Table 1). For example, Bailey *et al.* [1] showed integrons in the commensal *Escherichia coli* of healthy individuals with no exposure to antibiotics for six months. Most of the subjects carried at least one resistant strain in the low abundant population of the gut. In addition, these strains were diverse and resistant to 3–4 antibiotics.

Most of the current studies have been focused on integrons and AR resistance genes in easily cultivable bacteria [16]. A future challenge will therefore be to determine the contribution from the majority of the microbiota that cannot easily be cultivated. With the emergence of new sequencing techniques and single cell analytical approaches this will be a research field of high importance.

**Table 1:** Commensal microbiota as a source of antibiotic resistance (AR) genes in integrons.

Integron Class	Sampling Source	Integron Source	Resistance to antibiotic	Reference
I & II	Human feces	Commensal *E.coli*	Trimethoprim and aminoglycosides	[64]
I	Human feces	*Citrobacter freundii*	Aminoglycoside	[65]
II	Human feces	Commensal *E. coli*	Trimethoprim, streptomycin & spectinomycin	[1]
I	Human feces	*Enterobacteriaceae* and *Pseudomonadaceae*	Aminoglycoside, streptomycin and spectinomycin	[63]
I	Swine feces	Commensal *E coli*	Sulfamethoxazole, tetracycline and ampicillin	[66]
I	Captive wallabies feces	Metagenome	Spectinomycin, streptomycin and trimethoprim	[67]
I	Human feces	Commensal *E.coli*	Sulfamethoxazole, cefotaxime, gentamycin and ciprofloxacin.	[68]
ND*	Poultry and swine feces	Commensal *E. coli*	Tetracycline, sulfamethoxazole, quinolones and streptomycin	[69]
I	Human feces	Commensal *E.coli*	Streptomycin and Tetracycline	[70]
II	Human feces	Commensal *E. coli*	Streptomycin	[70]

* ND: Not determined.

## 5. Conclusion

Evidence is accumulating on intestinal microbiota playing a major role in the accumulation of resistance genes in their genomes. The bacteria in the gut are not only capable of acquiring resistance genes but also in aiding in the transmission of these genes to other bacteria in the gut. It is therefore important to assess the selective pressure that drives this spread of antibiotic resistance to humans.

## References

[1] Bailey J.K., Pinyon J.L., Anantham S., Hall R.M. (2010). Commensal Escherichia coli of healthy humans: a reservoir for antibiotic-resistance determinants. J. Med. Microbiol..

[2] Szmolka A., Nagy B. (2013). Multidrug resistant commensal Escherichia coli in animals and its impact for public health. FMICB.

[3] Jernberg C., Lofmark S., Edlund C., Jansson J.K. (2007). Long-term ecological impacts of antibiotic administration on the human intestinal microbiota. ISME J..

[4] Sullivan Å., Edlund C., Nord C.E. (2001). Effect of antimicrobial agents on the ecological balance of human microflora. Lancet. Infect. Dis..

[5] Administration F.D. Combating antibiotic resistance. http://www.fda.gov/downloads/ForConsumers/ConsumerUpdates/UCM143470.pdf.

[6] McKenna M. The Last resort. http://www.nature.com/news/antibiotic-resistance-the-last-resort-1.13426.

[7] Yong D., Toleman M.A., Giske C.G., Cho H.S., Sundman K., Lee K., Walsh T.R. (2009). Characterization of a New Metallo-β-Lactamase Gene, blaNDM-1, and a Novel Erythromycin Esterase Gene Carried on a Unique Genetic Structure in Klebsiella pneumoniae Sequence Type 14 from India. Antimicrob. Agents. Chemother..

[8] Salyers A.A., Gupta A., Wang Y. (2004). Human intestinal bacteria as reservoirs for antibiotic resistance genes. Trends. Microbiol..

[9] DiMarzio M., Shariat N., Kariyawasam S., Barrangou R., Dudley E.G. (2013). Antibiotic Resistance in Salmonella enterica Serovar Typhimurium Associates with CRISPR Sequence Type. Antimicrob. Agents. Chemother..

[10] Phillips I., Casewell M., Cox T., De Groot B., Friis C., Jones R., Nightingale C., Preston R., Waddell J. (2004). Does the use of antibiotics in food animals pose a risk to human health? A critical review of published data. J. Antimicrob. Chemother..

[11] Tian B., Fadhil N.H., Powell J.E., Kwong W.K., Moran N.A. (2012). Long-Term Exposure to Antibiotics Has Caused Accumulation of Resistance Determinants in the Gut Microbiota of Honeybees. mBio.

[12] Tavernise S. FDA restricts Antibiotic Use in lifestock. http://www.nytimes.com/2013/12/12/health/fda-to-phase-out-use-of-some-antibiotics-in-animals-raised-for-meat.html?pagewanted=all.

[13] Davies J., Davies D. (2010). Origins and evolution of antibiotic resistance. Microbiol. Mol. Biol. Rev..

[14] Khachatourians G.G. (1998). Agricultural use of antibiotics and the evolution and transfer of antibiotic-resistant bacteria. Can. Med. Assoc. J..

[15] Smillie C.S., Smith M.B., Friedman J., Cordero O.X., David L.A., Alm E.J. (2011). Ecology drives a global network of gene exchange connecting the human microbiome. Nature.

[16] Moore A.M., Patel S., Forsberg K.J., Wang B., Bentley G., Razia Y., Qin X., Tarr P.I., Dantas G. (2013). Pediatric fecal microbiota harbor diverse and novel antibiotic resistance genes. PLoS One.

[17] Ochman H., Lawrence J.G., Groisman E.A. (2000). Lateral gene transfer and the nature of bacterial innovation. Nature.

[18] Su J., Shi L., Yang L., Xiao Z., Li X., Yamasaki S. (2006). Analysis of integrons in clinical isolates of Escherichia coli in China during the last six years. FEMS Microbiol. Lett..

[19] Dobrindt U., Hacker J. (2001). Whole genome plasticity in pathogenic bacteria. Curr. Opin. Microbiol..

[20] Mazel D. (2006). Integrons: Agents of bacterial evolution. Nat. Rev. Microbiol..

[21] Frost L.S., Leplae R., Summers A.O., Toussaint A. (2005). Mobile genetic elements: the agents of open source evolution. Nat. Rev. Microbiol..

[22] Mazodier P., Davies J. (1991). Gene transfer between distantly related bacteria. Annu. Rev. Genet..

[23] Baharoglu Z., Bikard D., Mazel D. (2010). Conjugative DNA Transfer Induces the Bacterial SOS Response and Promotes Antibiotic Resistance Development through Integron Activation. PLoS Genet..

[24] Brisson-Noel A., Arthur M., Courvalin P. (1988). Evidence for natural gene transfer from gram-positive cocci to Escherichia coli. J. Bacteriol..

[25] Grohmann E., Muth G., Espinosa M. (2003). Conjugative plasmid transfer in gram-positive bacteria. Microbiol. Mol. Biol. Rev..

[26] Meng H., Zhang Z., Chen M., Su Y., Li L., Miyoshi S., Yan H., Shi L. (2011). Characterization and horizontal transfer of class 1 integrons in Salmonella strains isolated from food products of animal origin. Int. J. Food Microbiol..

[27] Kruse H., Sørum H. (1994). Transfer of multiple drug resistance plasmids between bacteria of diverse origins in natural microenvironments. Appl. Environ. Microbiol..

[28] Lester C.H., Frimodt-Moller N., Hammerum A.M. (2004). Conjugal transfer of aminoglycoside and macrolide resistance between Enterococcus faecium isolates in the intestine of streptomycin-treated mice. FEMS Microbiol. Lett..

[29] Domingues S., da Silva G.J., Nielsen K.M. (2012). Integrons: Vehicles and pathways for horizontal dissemination in bacteria. Mob. Genet. Elements..

[30] Domingues S., Harms K., Fricke W.F., Johnsen P.J., da Silva G.J., Nielsen K.M. (2012). Natural Transformation Facilitates Transfer of Transposons, Integrons and Gene Cassettes between Bacterial Species. PLoS Pathog..

[31] Davison J. (1999). Genetic exchange between bacteria in the environment. Plasmid.

[32] Kovalevskaya N.P. (2002). Mobile Gene Cassettes and Integrons. Mol. Biol..

[33] Fluit A.C., Schmitz F.J. (2004). Resistance integrons and super-integrons. Clin. Microbiol. Infect..

[34] Petersen A., Guardabassi L., Dalsgaard A., Olsen J.E. (2000). Class I integrons containing a dhfrI trimethoprim resistance gene cassette in aquatic Acinetobacter spp. FEMS Microbiol. Lett..

[35] Gallego L., Towner K.J. (2001). Carriage of class 1 integrons and antibiotic resistance in clinical isolates of Acinetobacter baumannii from northern Spain. J. Med. Microbiol..

[36] Chang C.Y., Chang L.L., Chang Y.H., Lee T.M., Chang S.F. (2000). Characterisation of drug resistance gene cassettes associated with class 1 integrons in clinical isolates of Escherichia coli from Taiwan, ROC. J. Med. Microbiol..

[37] Mazel D., Dychinco B., Webb V.A., Davies J. (2000). Antibiotic resistance in the ECOR collection: integrons and identification of a novel aad gene. Antimicrob. Agents Chemother..

[38] Casin I., Breuil J., Brisabois A., Moury F., Grimont F., Collatz E. (1999). Multidrug-resistant human and animal Salmonella typhimurium isolates in France belong predominantly to a DT104 clone with the chromosome- and integron-encoded beta-lactamase PSE-1. J. Infect. Dis..

[39] Orman B.E., Pineiro S.A., Arduino S., Galas M., Melano R., Caffer M.I., Sordelli D.O., Centron D. (2002). Evolution of multiresistance in nontyphoid salmonella serovars from 1984 to 1998 in Argentina. Antimicrob. Agents Chemother..

[40] Gonzalez G., Sossa K., Bello H., Dominguez M., Mella S., Zemelman R. (1998). Presence of integrons in isolates of different biotypes of Acinetobacter baumannii from Chilean hospitals. FEMS Microbiol. Lett..

[41] Hochhut B., Lotfi Y., Mazel D., Faruque S.M., Woodgate R., Waldor M.K. (2001). Molecular analysis of antibiotic resistance gene clusters in vibrio cholerae O139 and O1 SXT constins. Antimicrob. Agents Chemother..

[42] Mazel D., Dychinco B., Webb V.A., Davies J. (1998). A Distinctive Class of Integron in the Vibrio cholerae Genome. Science.

[43] Rowe-Magnus D.A., Guerout A.M., Ploncard P., Dychinco B., Davies J., Mazel D. (2001). The evolutionary history of chromosomal super-integrons provides an ancestry for multiresistant integrons. Proc. Natl. Acad. Sci. USA.

[44] Clark C.A., Purins L., Kaewrakon P., Focareta T., Manning P.A. (2000). The Vibrio cholerae O1 chromosomal integron. Microbiology.

[45] Guo X., Xia R., Han N., Xu H. (2011). Genetic diversity analyses of class 1 integrons and their associated antimicrobial resistance genes in Enterobacteriaceae strains recovered from aquatic habitats in China. Lett. Appl. Microbiol..

[46] Leverstein-van Hall M.A., Box A.T.A., Blok H.E.M., Paauw A., Fluit A.C., Verhoef J. (2002). Evidence of Extensive Interspecies Transfer of Integron-Mediated Antimicrobial Resistance Genes among Multidrug-Resistant Enterobacteriaceae in a Clinical Setting. J. Infect. Dis..

[47] Hocquet D., Llanes C., Thouverez M., Kulasekara H.D., Bertrand X., Plesiat P., Mazel D., Miller S.I. (2012). Evidence for induction of integron-based antibiotic resistance by the SOS response in a clinical setting. PLoS Pathog..

[48] Rensing C., Newby D.T., Pepper I.L. (2002). The role of selective pressure and selfish DNA in horizontal gene transfer and soil microbial community adaptation. Soil. Biol. Biochem..

[49] Gillings M.R., Xuejun D., Hardwick S.A., Holley M.P., Stokes H.W. (2009). Gene cassettes encoding resistance to quaternary ammonium compounds: a role in the origin of clinical class 1 integrons?. ISME J..

[50] Toleman M.A., Walsh T.R. (2011). Combinatorial events of insertion sequences and ICE in Gram-negative bacteria. FEMS Microbiol. Rev..

[51] Yatsunenko T., Rey F.E., Manary M.J., Trehan I., Dominguez-Bello M.G., Contreras M., Magris M., Hidalgo G., Baldassano R.N., Anokhin A.P. (2012). Human gut microbiome viewed across age and geography. Nature.

[52] Sekirov I., Russell S.L., Antunes L.C., Finlay B.B. (2010). Gut microbiota in health and disease. Physiol. Rev..

[53] Penders J., Thijs C., Vink C., Stelma F.F., Snijders B., Kummeling I., van den Brandt P.A., Stobberingh E.E. (2006). Factors influencing the composition of the intestinal microbiota in early infancy. Pediatrics.

[54] Jimenez E., Marin M.L., Martin R., Odriozola J.M., Olivares M., Xaus J., Fernandez L., Rodriguez J.M. (2008). Is meconium from healthy newborns actually sterile?. Res. Microbiol..

[55] Jimenez E., Fernandez L., Marin M.L., Martin R., Odriozola J.M., Nueno-Palop C., Narbad A., Olivares M., Xaus J., Rodriguez J.M. (2005). Isolation of commensal bacteria from umbilical cord blood of healthy neonates born by cesarean section. Curr. Microbiol..

[56] Satokari R., Gronroos T., Laitinen K., Salminen S., Isolauri E. (2009). Bifidobacterium and Lactobacillus DNA in the human placenta. Lett. Appl. Microbiol..

[57] Palmer C., Bik E.M., DiGiulio D.B., Relman D.A., Brown P.O. (2007). Development of the human infant intestinal microbiota. PLoS Biol..

[58] Avershina E., Storro O., Oien T., Johnsen R., Pope P., Rudi K. (2013). Major faecal microbiota shifts in composition and diversity with age in a geographically restricted cohort of mothers and their children. FEMS Microbiol. Ecol..

[59] Chung H., Pamp S.J., Hill J.A., Surana N.K., Edelman S.M., Troy E.B., Reading N.C., Villablanca E.J., Wang S., Mora J.R. (2012). Gut immune maturation depends on colonization with a host-specific microbiota. Cell.

[60] De Filippo C., Cavalieri D., Di Paola M., Ramazzotti M., Poullet J.B., Massart S., Collini S., Pieraccini G., Lionetti P. (2010). Impact of diet in shaping gut microbiota revealed by a comparative study in children from Europe and rural Africa. Proc. Natl. Acad. Sci. USA.

[61] Stephen A.M., Cummings J.H. (1980). The microbial contribution to human faecal mass. J. Med. Microbiol..

[62] Freilich S., Kreimer A., Meilijson I., Gophna U., Sharan R., Ruppin E. (2010). The large-scale organization of the bacterial network of ecological co-occurrence interactions. Nucleic. Acids. Res..

[63] Lévesque C., Piché L., Larose C., Roy P.H. (1995). PCR mapping of integrons reveals several novel combinations of resistance genes. Antimicrob. Agents Chemother..

[64] Lee J.C., Kang H.Y., Oh J.Y., Jeong J.H., Kim J., Seol S.Y., Cho D.T., Lee Y.C. (2006). Antimicrobial Resistance and Integrons Found in Commensal Escherichia coli Isolates from Healthy Humans. J. Bacteriol. Virol..

[65] Norskov-Lauritsen N., Sandvang D., Hedegaard J., Fussing V., Mortensen K.K., Sperling-Petersen H.U., Schonheyder H.C. (2001). Clonal origin of aminoglycoside-resistant Citrobacter freundii isolates in a Danish county. J. Med. Microbiol..

[66] Phongpaichit S., Liamthong S., Mathew A.G., Chethanond U. (2007). Prevalence of class 1 integrons in commensal Escherichia coli from pigs and pig farmers in Thailand. J. Food Prot..

[67] Power M.L., Emery S., Gillings M.R. (2013). Into the Wild: Dissemination of Antibiotic Resistance Determinants via a Species Recovery Program. PLoS ONE.

[68] Sepp E., Stsepetova J., Loivukene K., Truusalu K., Koljalg S., Naaber P., Mikelsaar M. (2009). The occurrence of antimicrobial resistance and class 1 integrons among commensal Escherichia coli isolates from infants and elderly persons. Ann. Clin. Microbiol. Antimicrob..

[69] Koo H.J., Woo G.J. (2012). Characterization of antimicrobial resistance of Escherichia coli recovered from foods of animal and fish origin in Korea. J. Food Prot..

[70] Skurnik D., Le Menac'h A., Zurakowski D., Mazel D., Courvalin P., Denamur E., Andremont A., Ruimy R. (2005). Integron-Associated Antibiotic Resistance and Phylogenetic Grouping of Escherichia coli Isolates from Healthy Subjects Free of Recent Antibiotic Exposure. Antimicrob. Agents Chemother..

